# Exploring non-linear distance metrics in the structure–activity space: QSAR models for human estrogen receptor

**DOI:** 10.1186/s13321-018-0300-0

**Published:** 2018-09-18

**Authors:** Ilya A. Balabin, Richard S. Judson

**Affiliations:** 1 Leidos, Inc., 109 TW Alexander Drive, MD N127-01, Research Triangle Park, NC 27711 USA; 2 US EPA, 109 TW Alexander Drive, ORD, NCCT, Research Triangle Park, NC 27711 USA

**Keywords:** Chemical space, Molecular similarity, Distance metrics, Structure–activity landscape, QSAR models, Human estrogen receptor

## Abstract

**Background:**

Quantitative structure-activity relationship (QSAR) models are important tools used in discovering new drug candidates and identifying potentially harmful environmental chemicals. These models often face two fundamental challenges: limited amount of available biological activity data and noise or uncertainty in the activity data themselves. To address these challenges, we introduce and explore a QSAR model based on custom distance metrics in the structure-activity space.

**Methods:**

The model is built on top of the k-nearest neighbor model, incorporating non-linearity not only in the chemical structure space, but also in the biological activity space. The model is tuned and evaluated using activity data for human estrogen receptor from the US EPA ToxCast and Tox21 databases.

**Results:**

The model closely trails the CERAPP consensus model (built on top of 48 individual human estrogen receptor activity models) in agonist activity predictions and consistently outperforms the CERAPP consensus model in antagonist activity predictions.

**Discussion:**

We suggest that incorporating non-linear distance metrics may significantly improve QSAR model performance when the available biological activity data are limited.
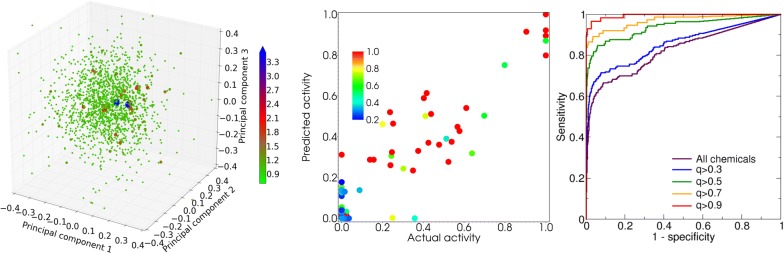

**Electronic supplementary material:**

The online version of this article (10.1186/s13321-018-0300-0) contains supplementary material, which is available to authorized users.

## Introduction

Identifying and understanding the connection between chemical structure and biological activity is a central problem in contemporary pharmacology and toxicology. Advances in such understanding could facilitate in silico discovery of novel drug candidates and give rise to more efficient methods for computational screening of environmental chemicals for potential adverse effects on human health [1, 2]. QSAR models address this problem by establishing structure–activity relationships from available chemical and biological data (training set) and using these relationships to estimate biological activities of other chemicals (evaluation set). In order to do so, QSAR models often utilize structure–activity landscapes, i.e., biological response surfaces in the structure–activity space reconstructed from the training set data [3]. The structure–activity landscapes are particularly useful for identifying chemical space domains where activity smoothly depends on structure (“rolling hills”) and those where small structural changes lead to significant changes in activity (“activity cliffs”) [4]. However, the limited size of typical training sets translates into the limited “resolution” of the reconstructed structure–activity landscapes: the latter only reveal net activity changes from one training set chemical to another but not details of the structure–activity relationship in-between these chemicals [5]. For example, if a training set only includes chemicals with similar activities, the reconstructed structure–activity landscape will be smooth, even though the actual structure–activity landscape may be rugged because of other chemicals with significantly different activities. In that case, the limited size of the training set may result in disappointing accuracy of QSAR model predictions [5]. Since activity cliffs are essential for specificity of many biological targets, most notably receptors, the limited amount of available activity data is a fundamental challenge that QSAR models face.

To address this challenge, we introduce and explore a QSAR model based on custom distance metrics in the structure-activity space. The distance metrics are designed to place higher (or lower, depending on the model parameters) weights on structurally close chemicals and chemicals with higher biological activities. We build our model on top of a simple approach that directly applies the similarity principle—the k-nearest neighbor (kNN) model [6]. Whereas the kNN model with non-Euclidean distances have been in use for decades [7], this, to the best of our knowledge, is the first attempt to incorporate non-linearity not only in the chemical structure space, but also in the biological activity space. We term this approach the generalized k-nearest neighbor (GkNN) model. Since we focus on the effects of the non-linearity of the distance metrics rather than the choice of a specific metric, we do not perform feature selection [8] but rather utilize conventional chemical fingerprints and similarity measures.

We evaluate the GkNN approach by building and tuning a model for human estrogen receptor (hER) activity using data from the US EPA ToxCast [9] and Tox21 [10] databases. Because of the critical regulatory role of the hER as a part of the endocrine system, the influence of chemicals on its activity has been extensively studied using a variety of methods such as molecular dynamics and docking [11, 12], CoMFA [13], pharmacophore-based QSAR modeling [14], and high-throughput screening [15]. We compare the performance of the GkNN-hER model with the recently developed CERAPP (Collaborative Estrogen Receptor Activity Prediction Project) consensus model built on top of 48 other classification and regression models [16].

## Methods

### Chemical and biological data

The training set included 1667 chemicals from the ToxCast database [9]. The training set chemicals were curated while they were prepared for the CERAPP collaboration; the curation procedure is described in the CERAPP article [16]. The chemicals had hER agonist, antagonist, and binding activity scores on the scale from 0.0 (inactive) to 1.0 (active). These activity scores were derived from a model that combined data from 18 in vitro hER assays using a variety of different cell types and readout technologies [2]. Because all assays yield some false positives and false negatives, we created a model to quantify our belief that the activity was “true” (i.e., it arose from interaction of the chemicals and the hER), or false (i.e., it arose from some form of technology interference or simple experimental noise) [2]. The activity value for a chemical represents an estimate of potency (the higher the value, the lower the concentration of the chemical that is required to activate the receptor), but also a certainty that the chemical actually interacts with hER [2]. Chemicals with low activity values (e.g., below 0.1) have a higher chance of being false positives than do chemicals with values well above this cutoff. To reduce the uncertainty, a small number of chemicals with activity values between 0.01 and 0.1 was removed from the training set.

The evaluation set included 7221 chemicals from the CERAPP database [10] with AC50, IC50, and/or other hER activity measures reported in the literature [16] (see Additional file 1: Fig. S1). Agonist and antagonist activity scores on the scale from 0.0 to 1.0 for these chemicals were estimated from their AC50 values that constituted the vast majority of all activity data (39,804 out of 44,641 records for agonist activity) and the dependence obtained from the training set [9]. A small number of chemicals with missing AC50 data were not included in model evaluation. For each chemical, activity scores from different sources were averaged. In this larger dataset from Tox21 and the open literature, we observed the same lack of consistency from one assay to another (or one lab to another) in activity, and the range of values from 0.0 to 1.0 again represents a combination of estimated potency (higher values are more potent) and certainty of a true interaction with hER (higher values are more certain to be true actives).

In addition to the entire evaluation set, calculations were performed with its subsets that included more than 3, 5, 7, or 9 consistent activity sources per chemical, respectively. Consistent means that the majority call (active or inactive) had to occur in at least 80% of cases for a chemical. As chemicals required more consistent data (either positive or negative), the quality of the biological data increased, but the number of chemicals decreased.

### Structure–activity space

To visualize positions of the training set and evaluation set chemicals in the chemical structure space, we performed principal component analysis (PCA) on the fingerprints of the training set chemicals. The analysis was performed independently for Morgan and Indigo full fingerprints, and positions of the chemicals were described by their projections on the first three eigenvectors. In addition, relative positions of the chemicals were characterized by the distributions of pairwise molecular similarities (analogs of the radial distribution function commonly used in statistical mechanics) [17, 18]. To characterize how much positions of chemicals in the chemical structure space depend on the choice of the specific fingerprint, we compiled lists of nearest neighbors for each training set chemical using Morgan and Indigo full fingerprints, respectively.

The extent of ruggedness of the structure–activity landscape was described by the structure–activity landscape index [3] $SALI_{ij} = \left| {A_{i} - A_{j} } \right|/\left( {1 - S_{ij} } \right)$, where $A_{i}$ is the activity score of chemical $i$ and $S_{ij}$ is the similarity between chemicals $i$ and $j$. The distribution of the pairwise SALI values characterized the entire structure–activity landscape, whereas the maximum value per chemical $\mathop {\hbox{max} }\nolimits_{\text{j}} \left( {SALI_{ij} } \right)$ identified specific chemicals that form activity cliffs.

### GkNN model

The model estimates biological activity of a chemical as a non-linear weighted average over activities of $k$ most similar chemicals from the training set:

1 $$A_{i} = \left( {\frac{{\mathop \sum \nolimits_{j}^{k} A_{j}^{x} S_{ij}^{y} }}{{\mathop \sum \nolimits_{j}^{k} S_{ij}^{y} }}} \right)^{1/x} ,$$

where $A_{j}$ is the activity score of chemical $j$ and $S_{ij}$ is the molecular similarity between chemicals $i$ and $j$. The activity scores vary continuously in the range from 0.0 (inactive) to 1.0 (active), and a chemical is classified as active or inactive depending on whether its activity score exceeded a specified cutoff. The similarities vary continuously in the range from 0.0 to 1.0. The similarity to the closest chemical from the training set $q_{i} = \mathop { \hbox{max} }\nolimits_{j} \left( {S_{ij} } \right)$ characterizes the confidence in the estimate. Tunable parameters $x$ and $y$ characterize non-linearity in the biological activity space and the chemical structure space, respectively.

The GkNN model was compared with three other variations of kNN models suggested earlier [19]:

2 $$A_{i} = \frac{1}{k}\mathop \sum \limits_{j}^{k} A_{j} ,$$

3 $$A_{i} = \varPi_{j}^{k} A_{j}^{{{\raise0.7ex\hbox{$1$} \!\mathord{\left/ {\vphantom {1 k}}\right.\kern-0pt} \!\lower0.7ex\hbox{$k$}}}} ,$$

4 $$A_{i} = \frac{{\mathop \sum \nolimits_{j}^{k} A_{j} { \exp }\left( { - xd_{ij} } \right)}}{{\mathop \sum \nolimits_{j}^{k} { \exp }\left( { - xd_{ij} } \right)}}.$$

These models are based on arithmetic averaging of the nearest neighbor activities (Eq. 2), geometric averaging of these activities (Eq. 3), and exponential averaging of these activities weighted by distances to the neighbors in the chemical structure space (Eq. 4). In the exponential model, we assumed that the distances are related with molecular similarities as $d_{ij} = 1/S_{ij} - 1$ and added a tunable parameter X that varied between 0.1 and 10. Molecular similarities were calculated using MACCS keys, Daylight, Morgan, or Indigo full fingerprints and Tanimoto similarity [20]. Calculations with Morgan fingerprints folded to 1024 bits and 16,384 bits, respectively, produced nearly identical results, indicating that increasing the fingerprint folding size beyond about 1000 bits has negligible influence on the performance of QSAR models. Whereas the obtained activity estimates were qualitatively similar for all fingerprints, using Morgan or Indigo full fingerprints consistently resulted in more accurate estimates.

### Parameter tuning and evaluation

The accuracy of agonist, antagonist, and binding activity estimates obtained using the GkNN model and other models was characterized by the following metrics

- Sensitivity (true positive rate): $TPR = TP/\left( {TP + FN} \right)$
- Specificity (true negative rate): $TNR = TN/\left( {TN + FP} \right)$
- Balanced accuracy (non-error rate): $NER = 0.5*\left( {TPR + TNR} \right)$
- Accuracy: $$A = \left( {TP + TN} \right)/\left( {TP + FP + FN + TN} \right)$$
- Precision (positive predicted value): $PPV = TP/\left( {TP + FP} \right)$
- Negative predicted value: $NPV = TN/\left( {TN + FN} \right)$
- ROC AUC

Here, TP, FP, FN, and TN indicate the numbers of true positive, false positive, false negative, and true negative evaluations, respectively. These numbers were obtained by converting continuous activity estimates to binary classes using the same activity threshold of 0.1 that was used for the training set.

To identify the values of parameters $k$, $x$, and $y$ that yield the most accurate estimates, leave-one-out cross-validation calculations for the training set were performed with every combination of the model parameters from the following lists (2560 combinations total):

$$k = 1, 2, 3, 5, 7, 10, 15, 20, 30, 50$$

$$x = 0.0, 0.1, 0.2, 0.3, 0.5, 0.7, 1.0, 1.5, 2.0, 3.0, 5.0, 7.0, 10.0, 15.0, 20.0, 30.0, 50.0$$

$$y = 0.0, 0.1, 0.2, 0.3, 0.5, 0.7, 1.0, 1.5, 2.0, 3.0, 5.0, 7.0, 10.0, 15.0, 20.0, 30.0, 50.0.$$

Since different parameterizations of the model were found to maximize different accuracy metrics, parameterizations were ranked by the score defined as the product of balanced accuracy, accuracy, and ROC AUC. Parameterizations that maximize this score were also found to result in nearly maximum values of individual accuracy metrics, indicating that this score provides a robust characteristics of the QSAR model accuracy. Optimal parameterizations were independently identified for agonist, antagonist, and binding activities.

Model evaluation included estimating agonist and antagonist activities for the evaluation set chemicals. The evaluation did not include estimating binding activities, since their values for the evaluation set chemicals were not derived from AC50 data. The evaluation was performed using the optimal parameter combinations identified by cross-validation.

### Software implementation

The GkNN model was implemented as a set of standalone Python scripts. Chemical fingerprints and molecular similarities were calculated using open source cheminformatics toolkits RDKit [21] and Indigo [22], activity estimates were obtained using NumPy toolkit [23], and accuracy metrics were calculated using Scikit-learn toolkit [24].

## Results and discussion

### Chemical structure space

**Fig. 1:**
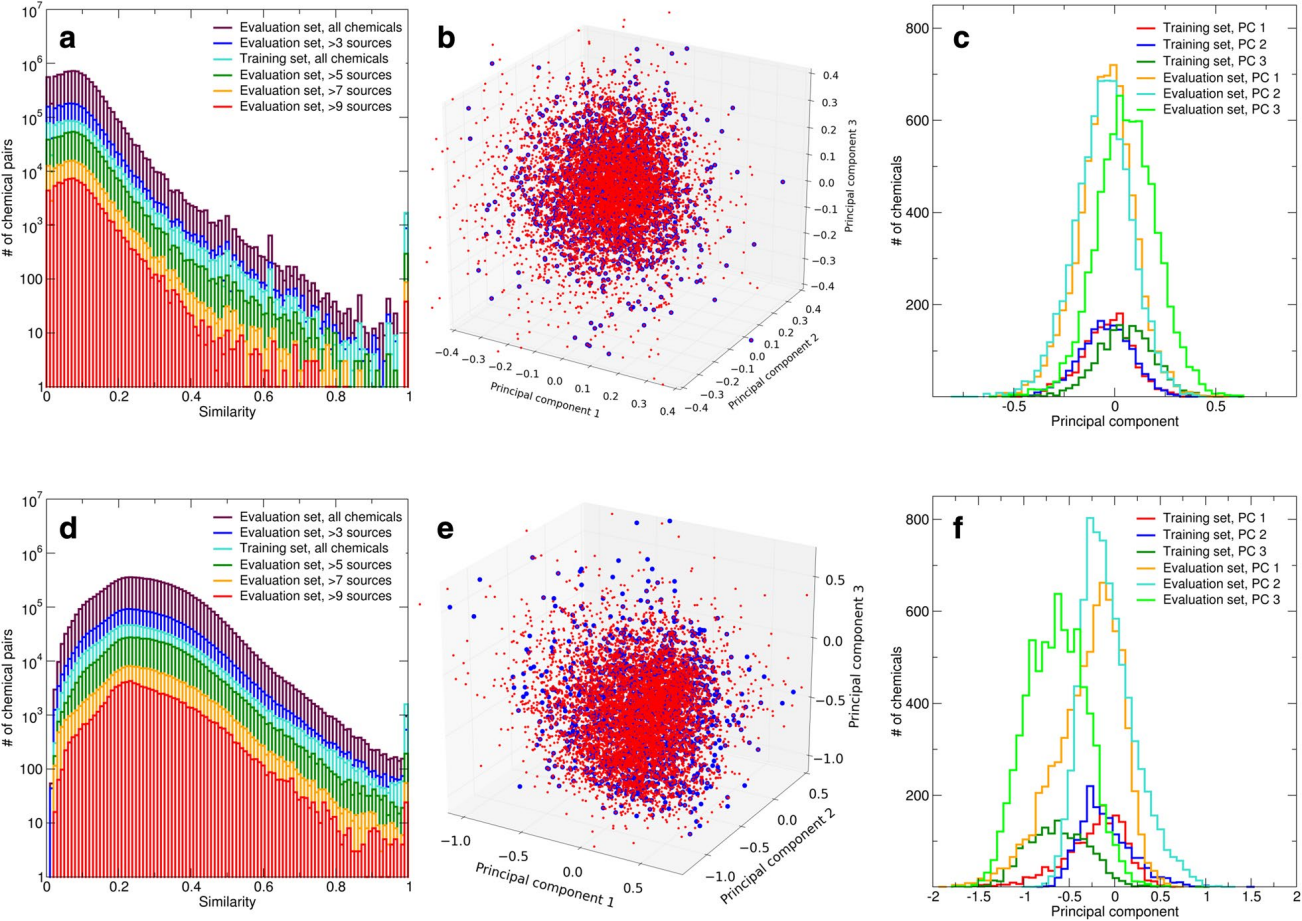
Chemical space domains occupied by the training set and the evaluation set. Results shown in panels **a**–**c** were calculated using Morgan fingerprints and Tanimoto similarity, and results shown in panels **d**–**f** were calculated using Indigo full fingerprints and Tanimoto similarity, respectively. Panels **a**, **d** distributions of pairwise molecular similarities (overlaid plots). Panels **b**, **e** projections of the training set (blue) and the evaluation set (red) on the 3D space formed by the first three eigenvectors of the training set self-similarity matrix. Panels C and F: distributions of the training and evaluation sets along each of the first three eigenvectors

Figure 1 indicates that the training set chemicals and the evaluation set chemicals occupy similar domains of the chemical structure space. Chemicals from both sets form approximately Gaussian distributions with a common center and similar shape (the widths of the evaluation set are slightly larger than those of the training set). Whereas using Morgan fingerprints and Indigo full fingerprints results in significantly different absolute similarity values, the above observations hold for the both fingerprints, suggesting that structure–activity relationships inferred from the training set are likely to hold for the evaluation set. This conclusion is further supported by the distributions of pairwise molecular similarities calculated using Indigo full and Morgan fingerprints (Fig. 1) and MACCS keys (Additional file 1: Fig. S2).

How sensitive is the “neighborhood” of a chemical in the chemical structure space to the choice of the molecular fingerprint? To address this question, we compiled neighbor lists for each chemical in the training set using Morgan and Indigo full fingerprints, respectively. We found that these lists significantly overlap, although the order of neighbors in the two lists is essentially different. As such, the overall arrangement of chemicals in the chemical structure space may be robust to the choice of the fingerprint, whereas the order of nearest neighbors for each chemical is fingerprint-sensitive.

### Structure–activity landscape

**Fig. 2:**
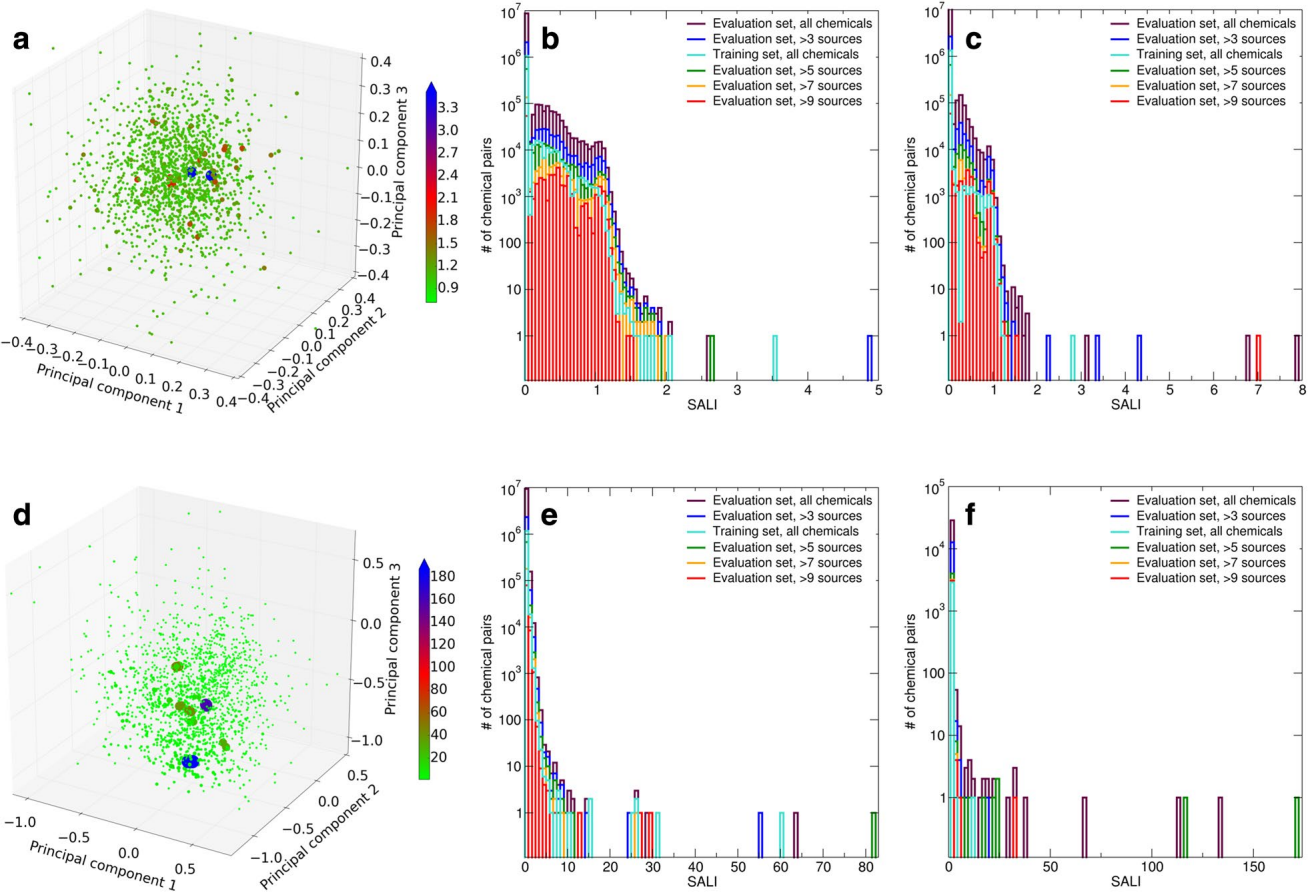
Structure–activity landscapes of the training set and the evaluation set. Results shown in panels **a**–**c** were calculated using Morgan fingerprints, and results shown in panels **d**–**f** were calculated using Indigo full fingerprints, respectively. Panels **a**, **d** maximum SALI values per chemical for agonist activities of the training set chemicals projected on the 3D space described in Fig. 1. Panels **b**, **e** distributions of the SALI values for agonist activities (overlaid plots). Panels **c**, **f** distributions of the SALI values for antagonist activities (overlaid plots)

Panels A and D in Fig. 2 show that the hER structure-agonist activity landscape obtained from the training set chemicals is mostly smooth, except for a few cliffs that arise from pairs of chemicals with similar structures but significantly different activities. Panels B and E in Fig. 2 along with Additional file 1: Fig. S3 support this conclusion, indicating that the structure-agonist activity landscapes for the training set, the evaluation set, and subsets of the evaluation set are characterized by relatively small SALI values, and higher SALI values that indicate activity cliffs are rare exceptions. Similarly, panels C and F in Fig. 2 along with Additional file 1: Fig. S3 show that the hER structure-antagonist activity landscape is even more smooth than that for agonist activity, in part because the number of active antagonist chemicals (9) was much smaller than the number of active agonist ones (80). Importantly, even though Morgan fingerprints, Indigo full fingerprints, and MACCS keys result in significantly different molecular similarity values and therefore different absolute SALI values, the above conclusions hold for the all fingerprints.

For the evaluation set, increasing the minimum number of sources per chemical reduces the number of chemicals and thereby increases the average distance among them in the chemical structure space. This has the effect of smoothing the structure–activity landscape, as indicated by the elimination of the larger SALI values. For chemicals with more than 9 activity sources, differences in activities are close to either 0.0 or 1.0, suggesting that these chemicals are either clearly active or clearly inactive. We therefore conclude that the full hER structure–activity landscape is more rugged than those reconstructed from the available chemical sets. As discussed above, this ruggedness may be key factor that limits the accuracy of QSAR models.

### Optimal parameters

Table 1 shows the accuracy metrics for the tuned GkNN model and the arithmetic, geometric, and exponential averaging kNN models. In all cross-validation calculations, the geometric averaging kNN model was consistently the least accurate one, whereas the arithmetic averaging kNN model performed considerably better, and the exponential averaging kNN model provided further improvement in accuracy. These results are consistent with the earlier calculations of melting point using these models [19]. The tuned GkNN model was found to provide an increase in balanced accuracy over the exponential averaging kNN model.

**Table 1:** Accuracy metrics for agonist, antagonist, and binding activity cross-validation

Activity	# chemicals	Model and parameters	Sensitivity	Specificity	Bal accuracy	Accuracy	ROC AUC	Score
Agonist	1538	Morgan kNN arithm k = 10	0.63	0.98	0.80	*0.96*	0.91	0.70
Agonist	1538	Morgan kNN geom k = 2	0.40	*0.99*	0.70	*0.96*	0.73	0.49
Agonist	1538	Morgan kNN exp k = 10 X = 1.5	0.69	0.97	0.83	*0.96*	*0.92*	*0.73*
Agonist	1538	Morgan GkNN k = 10 X = 1 Y = 1	0.63	0.98	0.80	*0.96*	*0.92*	0.70
Agonist	1538	Morgan GkNN k = 10 X = 1 Y = 3	0.66	0.97	0.82	*0.96*	*0.92*	*0.72*
Agonist	1538	Morgan GkNN k = 10 X = 1.5 Y = 3	*0.74*	0.95	*0.84*	0.94	*0.92*	*0.72*
Agonist	1538	Morgan GkNN k = 20 X = 1.5 Y = 5	*0.75*	0.95	*0.85*	0.94	0.91	*0.73*
Antagonist	1645	Morgan kNN arithm k = 3	0.44	*1.00*	0.72	*1.00*	0.70	0.51
Antagonist	1645	Morgan kNN geom k = 3	0.00	*1.00*	0.50	0.99	0.50	0.25
Antagonist	1645	Morgan kNN exp k = 3 X = 1.5	0.44	*1.00*	0.72	*1.00*	0.70	0.51
Antagonist	1645	Indigo kNN arithm k = 10	0.22	*1.00*	0.61	0.99	*0.73*	0.44
Antagonist	1645	Indigo kNN geom k = 10	0.00	*1.00*	0.50	0.99	0.50	0.25
Antagonist	1645	Indigo kNN exp k = 10 X = 1.5	0.44	*1.00*	0.72	0.99	*0.73*	*0.53*
Antagonist	1645	Indigo GkNN k = 10 X = 3 Y = 7	*0.56*	0.98	*0.77*	0.98	*0.73*	*0.55*
Antagonist	1645	Indigo GkNN k = 10 X = 5 Y = 15	*0.56*	0.98	*0.77*	0.98	*0.73*	*0.55*
Binding	1529	Morgan kNN arithm k = 10	0.63	0.98	0.80	*0.96*	*0.90*	0.69
Binding	1529	Morgan kNN geom k = 2	0.43	*0.99*	0.71	*0.96*	0.74	0.50
Binding	1529	Morgan kNN exp k = 10 X = 1.5	0.69	0.97	0.83	0.95	*0.90*	*0.71*
Binding	1529	Morgan GkNN k = 10 X = 1 Y = 1	0.63	0.98	0.80	*0.96*	*0.90*	0.69
Binding	1529	Morgan GkNN k = 10 X = 1 Y = 3	0.66	0.97	0.82	0.95	*0.90*	*0.70*
Binding	1529	Morgan GkNN k = 10 X = 1.5 Y = 3	*0.73*	0.94	*0.84*	0.93	*0.90*	*0.70*
Binding	1529	Morgan GkNN k = 20 X = 1.5 Y = 5	*0.75*	0.95	*0.85*	0.94	0.89	*0.71*

“kNN arithm”, “kNN geom”, and “kNN exp” indicate the kNN models with the arithmetic, geometric, and exponential averaging, respectively. The cumulative score shown in the last column is the product of balanced accuracy, accuracy, and ROC AUC. Italic font indicates accuracy metric values that exceed those for the CERAPP consensus model

For agonist and binding activity, the most accurate estimates were obtained by using Morgan fingerprints with $k = 10$. Increasing the values of the GkNN model parameters X and Y from $1.0$ to $1.5$ and $3.0$, respectively, resulted in a small increase in balanced accuracy and had no significant effect on ROC AUC. A similar increase in balanced accuracy was observed when the value of the exponential kNN model parameter X increased from $1.0$ to $1.5$. Interestingly, all models (except the geometric kNN model that was consistently much less accurate than the others) performed almost as well when using Indigo fingerprints with $k = 7$ and the same values of parameters X and, for the GkNN model, Y. Using Daylight fingerprints or MACCS keys resulted in a significantly lower performance (see Additional file 1: Table S1).

For antagonist activity, using Indigo fingerprints with k = 10 resulted in the most accurate estimates. The exponential kNN model provided an improvement in balanced accuracy over the arithmetic kNN model. Using the exponential model with Morgan fingerprints and $k = 3$ resulted in similar outcome. Still, the highest balanced accuracy gain was achieved by using the GkNN model with Indigo fingerprints, $k = 10$, and two combinations of the other parameters: $X = 3$, $Y = 7$ and $X = 5$, $Y = 15$, respectively. We suggest that the higher optimum values of $X$ and $Y$ for agonist activity calculations arise from the significantly smaller number of the agonist active chemicals, as discussed above.

Notably, multiple parameter combinations resulted in nearly identical accuracy in cross-validation as well as evaluation, indicating that the model parameters are not completely independent. Indeed, parameter $k$ that controls the number of relevant nearest neighbors and parameter $Y$ that weights contributions from these neighbors both influence the distance in the chemical structure space where the similarity principle is assumed to break down. Accordingly, simultaneously increasing parameters $k$ and $Y$ was found to have minor effect on the GkNN model estimates compared to changing one of those parameters. The above conclusions held when using Indigo full fingerprints as well, although the optimal parameter values in that case were different.

The optimal value of parameter $X > 1$ suggests that lower (but non-zero) biological activity estimates obtained from assay data might be not as reliable as higher activity estimates, consistent with the analysis of the assay data [2] and the activity distributions for different numbers of literature sources (see Additional file 1: Fig. S4). The optimal value of parameter $Y > 1$ indicates that the structure–activity principle is more likely to hold at closer distances in the chemical structure space, supporting the conclusion that the full hER structure–activity landscape is more rugged than the one reconstructed from the training set and/or the evaluation set.

### Model performance

Tables 2 and 3 summarize the accuracy of agonist and antagonist activity estimates for the evaluation set chemicals obtained by using the kNN models, the GkNN model, and the CERAPP consensus model [16]. As in cross-validation, the geometric kNN model yielded the least accurate estimates, and the arithmetic kNN model performed considerably better but not as well as the exponential kNN model or the GkNN model. In the agonist activity estimates (Table 2), the latter two performed on par with each other. They both closely trailed the CERAPP consensus model in ROC AUC and slightly outperformed it in balanced accuracy for chemicals with 5–9 activity sources. In most antagonist activity estimates (Table 3), the exponential kNN model was on par with the CERAPP consensus model in balanced accuracy and slightly outperformed it in ROC AUC, whereas the GkNN model consistently outperformed the both. Notably, the improvement in balanced accuracy provided by the GkNN model over the exponential kNN model was higher for chemicals with larger numbers of activity sources.

**Table 2:** Accuracy metrics for agonist activity evaluation with different numbers of activity sources per chemical

# sources	# chemicals	Model and parameters	Sensitivity	Specificity	Bal accuracy	Accuracy	ROC AUC	Score
1	6197	CERAPP consensus	*0.71*	0.95	*0.83*	0.94	*0.85*	*0.67*
1	6197	Morgan kNN arithm k = 10	0.55	*0.96*	0.75	0.94	0.82	0.58
1	6197	Morgan kNN geom k = 2	0.38	*0.99*	0.69	*0.97*	0.72	0.48
1	6197	Morgan kNN exp k = 10 X = 1.5	0.59	*0.97*	0.78	*0.95*	0.83	0.61
1	6197	Morgan GkNN k = 10 X = 1 Y = 1	0.58	*0.96*	0.77	0.94	0.82	0.59
1	6197	Morgan GkNN k = 10 X = 1 Y = 3	0.59	*0.97*	0.78	*0.95*	0.83	0.61
1	6197	Morgan GkNN k = 10 X = 1.5 Y = 3	0.64	0.93	0.78	0.92	0.82	0.59
1	6197	Morgan GkNN k = 20 X = 1.5 Y = 5	0.64	0.94	0.79	0.93	0.83	0.61
3	1553	CERAPP consensus	*0.93*	0.94	*0.94*	0.94	*0.98*	*0.87*
3	1553	Morgan kNN arithm k = 10	0.77	*0.95*	0.86	*0.94*	0.94	0.76
3	1553	Morgan kNN geom k = 2	0.57	*0.99*	0.78	*0.97*	0.80	0.60
3	1553	Morgan kNN exp k = 10 X = 1.5	0.82	*0.97*	0.89	*0.96*	0.95	0.81
3	1553	Morgan GkNN k = 10 X = 1 Y = 1	0.82	*0.96*	0.89	*0.95*	0.94	0.79
3	1553	Morgan GkNN k = 10 X = 1 Y = 3	0.83	*0.97*	0.90	*0.96*	0.95	0.82
3	1553	Morgan GkNN k = 10 X = 1.5 Y = 3	0.88	0.93	0.90	0.92	0.95	0.79
3	1553	Morgan GkNN k = 20 X = 1.5 Y = 5	0.88	*0.94*	0.91	*0.94*	0.94	0.80
5	456	CERAPP consensus	*0.96*	0.93	*0.94*	0.94	*0.99*	*0.88*
5	456	Morgan kNN arithm k = 10	0.81	*0.94*	0.88	0.93	0.94	0.77
5	456	Morgan kNN geom k = 2	0.68	*1.00*	0.84	*0.96*	0.86	0.69
5	456	Morgan kNN exp k = 10 X = 1.5	0.92	*0.97*	*0.94*	*0.96*	0.96	0.87
5	456	Morgan GkNN k = 10 X = 1 Y = 1	0.89	*0.95*	0.92	*0.94*	0.95	0.82
5	456	Morgan GkNN k = 10 X = 1 Y = 3	0.92	*0.97*	*0.94*	*0.96*	0.96	0.87
5	456	Morgan GkNN k = 10 X = 1.5 Y = 3	0.94	0.92	0.93	0.92	0.96	0.82
5	456	Morgan GkNN k = 20 X = 1.5 Y = 5	0.94	*0.95*	*0.94*	*0.95*	0.96	0.86
7	128	CERAPP consensus	*0.95*	0.95	*0.95*	0.95	*1.00*	0.90
7	128	Morgan kNN arithm k = 10	0.88	*0.98*	0.93	*0.95*	0.95	0.84
7	128	Morgan kNN geom k = 2	0.76	*1.00*	0.88	0.94	0.90	0.74
7	128	Morgan kNN exp k = 10 X = 1.5	0.91	*0.99*	*0.95*	*0.97*	0.96	0.89
7	128	Morgan GkNN k = 10 X = 1 Y = 1	0.94	*0.99*	*0.97*	*0.98*	0.96	0.90
7	128	Morgan GkNN k = 10 X = 1 Y = 3	0.94	*1.00*	*0.97*	*0.98*	0.97	*0.92*
7	128	Morgan GkNN k = 10 X = 1.5 Y = 3	0.94	0.91	0.93	0.92	0.96	0.82
7	128	Morgan GkNN k = 20 X = 1.5 Y = 5	0.94	*0.97*	*0.95*	*0.96*	0.97	0.89
9	57	CERAPP consensus	*0.92*	1.00	*0.96*	*0.97*	*1.00*	*0.93*
9	57	Morgan kNN arithm k = 10	0.79	1.00	0.89	0.93	0.93	0.78
9	57	Morgan kNN geom k = 2	0.79	1.00	0.89	0.93	0.92	0.77
9	57	Morgan kNN exp k = 10 X = 1.5	0.84	1.00	0.92	0.95	0.94	0.82
9	57	Morgan GkNN k = 10 X = 1 Y = 1	0.84	1.00	0.92	0.95	0.94	0.82
9	57	Morgan GkNN k = 10 X = 1 Y = 3	0.84	1.00	0.92	0.95	0.94	0.82
9	57	Morgan GkNN k = 10 X = 1.5 Y = 3	0.89	0.92	0.91	0.91	0.94	0.78
9	57	Morgan GkNN k = 20 X = 1.5 Y = 5	0.89	0.97	0.93	0.95	0.94	0.84

“kNN arithm”, “kNN geom”, and “kNN exp” indicate the kNN models with the arithmetic, geometric, and exponential averaging, respectively. The cumulative score shown in the last column is the product of balanced accuracy, accuracy, and ROC AUC. Italic font indicates accuracy metric values that exceed those for the CERAPP consensus model

**Table 3:** Accuracy metrics for antagonist activity evaluation with different numbers of activity sources per chemical

# sources	# chemicals	Model and parameters	Sensitivity	Specificity	Bal Accuracy	Accuracy	ROC AUC	Score
1	6533	CERAPP consensus	*0.15*	0.91	0.53	0.88	0.55	0.26
1	6533	Morgan kNN arithm k = 3	0.04	0.99	0.52	*0.95*	0.53	0.26
1	6533	Morgan kNN geom k = 3	0.00	*1.00*	0.50	*0.96*	0.51	0.24
1	6533	Morgan kNN exp k = 3 X = 1.5	0.04	0.99	0.52	*0.95*	0.53	0.26
1	6533	Indigo kNN arithm k = 10	0.04	0.99	0.52	*0.95*	*0.57*	*0.28*
1	6533	Indigo kNN geom k = 10	0.00	*1.00*	0.50	*0.96*	0.50	0.24
1	6533	Indigo kNN exp k = 10 X = 1.5	0.05	0.99	0.52	*0.95*	*0.57*	*0.28*
1	6533	Indigo GkNN k = 10 X = 3 Y = 7	0.10	0.98	*0.54*	*0.94*	*0.57*	*0.29*
1	6533	Indigo GkNN k = 10 X = 5 Y = 15	0.10	0.98	*0.54*	*0.94*	*0.57*	*0.29*
3	1707	CERAPP consensus	0.17	0.90	0.53	0.87	0.58	0.27
3	1707	Morgan kNN arithm k = 3	0.09	0.99	0.54	0.95	0.57	*0.29*
3	1707	Morgan kNN geom k = 3	0.00	*1.00*	0.50	0.95	0.53	0.25
3	1707	Morgan kNN exp k = 3 X = 1.5	0.10	*1.00*	0.55	*0.96*	0.57	*0.30*
3	1707	Indigo kNN arithm k = 10	0.12	*1.00*	0.56	*0.96*	*0.65*	*0.35*
3	1707	Indigo kNN geom k = 10	0.00	*1.00*	0.50	0.95	0.50	0.24
3	1707	Indigo kNN exp k = 10 X = 1.5	0.14	*1.00*	0.57	*0.96*	*0.65*	*0.36*
3	1707	Indigo GkNN k = 10 X = 3 Y = 7	*0.18*	0.99	*0.58*	0.95	*0.65*	*0.36*
3	1707	Indigo GkNN k = 10 X = 5 Y = 15	*0.18*	0.99	*0.58*	0.95	*0.65*	*0.36*
5	431	CERAPP consensus	*0.24*	0.89	0.56	0.84	*0.67*	0.32
5	431	Morgan kNN arithm k = 3	0.14	0.99	0.56	0.93	0.61	0.32
5	431	Morgan kNN geom k = 3	0.00	*1.00*	0.50	0.93	0.52	0.24
5	431	Morgan kNN exp k = 3 X = 1.5	0.17	*1.00*	*0.58*	*0.94*	0.61	*0.34*
5	431	Indigo kNN arithm k = 10	0.10	*1.00*	0.55	*0.94*	0.65	*0.33*
5	431	Indigo kNN geom k = 10	0.00	*1.00*	0.50	0.93	0.50	0.23
5	431	Indigo kNN exp k = 10 X = 1.5	0.10	*1.00*	0.55	*0.94*	0.65	*0.33*
5	431	Indigo GkNN k = 10 X = 3 Y = 7	0.17	0.99	*0.58*	0.93	0.65	*0.35*
5	431	Indigo GkNN k = 10 X = 5 Y = 15	0.17	0.99	*0.58*	0.93	0.65	*0.35*
7	103	CERAPP consensus	*0.31*	0.91	0.61	0.84	0.67	0.34
7	103	Morgan kNN arithm k = 3	0.23	0.98	0.60	0.88	0.68	*0.36*
7	103	Morgan kNN geom k = 3	0.00	*1.00*	0.50	0.87	0.54	0.24
7	103	Morgan kNN exp k = 3 X = 1.5	0.23	*1.00*	0.62	*0.90*	0.68	*0.38*
7	103	Indigo kNN arithm k = 10	0.08	*1.00*	0.54	0.88	0.79	*0.38*
7	103	Indigo kNN geom k = 10	0.00	*1.00*	0.50	0.87	0.50	0.22
7	103	Indigo kNN exp k = 10 X = 1.5	0.15	0.98	0.57	0.87	*0.80*	*0.39*
7	103	Indigo GkNN k = 10 X = 3 Y = 7	0.23	0.98	0.60	0.88	*0.80*	*0.43*
7	103	Indigo GkNN k = 10 X = 5 Y = 15	0.31	0.99	*0.65*	*0.90*	*0.80*	*0.47*
9	46	CERAPP consensus	*0.40*	*1.00*	*0.70*	*0.87*	0.73	*0.44*
9	46	Morgan kNN arithm k = 3	0.30	0.97	0.64	0.83	0.73	0.38
9	46	Morgan kNN geom k = 3	0.00	*1.00*	0.50	0.78	0.55	0.22
9	46	Morgan kNN exp k = 3 X = 1.5	0.30	*1.00*	0.65	0.85	0.73	0.40
9	46	Indigo kNN arithm k = 10	0.10	*1.00*	0.55	0.80	0.79	0.35
9	46	Indigo kNN geom k = 10	0.00	*1.00*	0.50	0.78	0.50	0.20
9	46	Indigo kNN exp k = 10 X = 1.5	0.20	0.97	0.59	0.80	0.79	0.37
9	46	Indigo GkNN k = 10 X = 3 Y = 7	0.30	0.97	0.64	0.83	*0.80*	0.42
9	46	Indigo GkNN k = 10 X = 5 Y = 15	0.40	1.00	*0.70*	*0.87*	*0.80*	0.49

“kNN arithm”, “kNN geom”, and “kNN exp” indicate the kNN models with the arithmetic, geometric, and exponential averaging, respectively. The cumulative score shown in the last column is the product of balanced accuracy, accuracy, and ROC AUC. Italic font indicates accuracy metric values that exceed those for the CERAPP consensus model

**Fig. 3:**
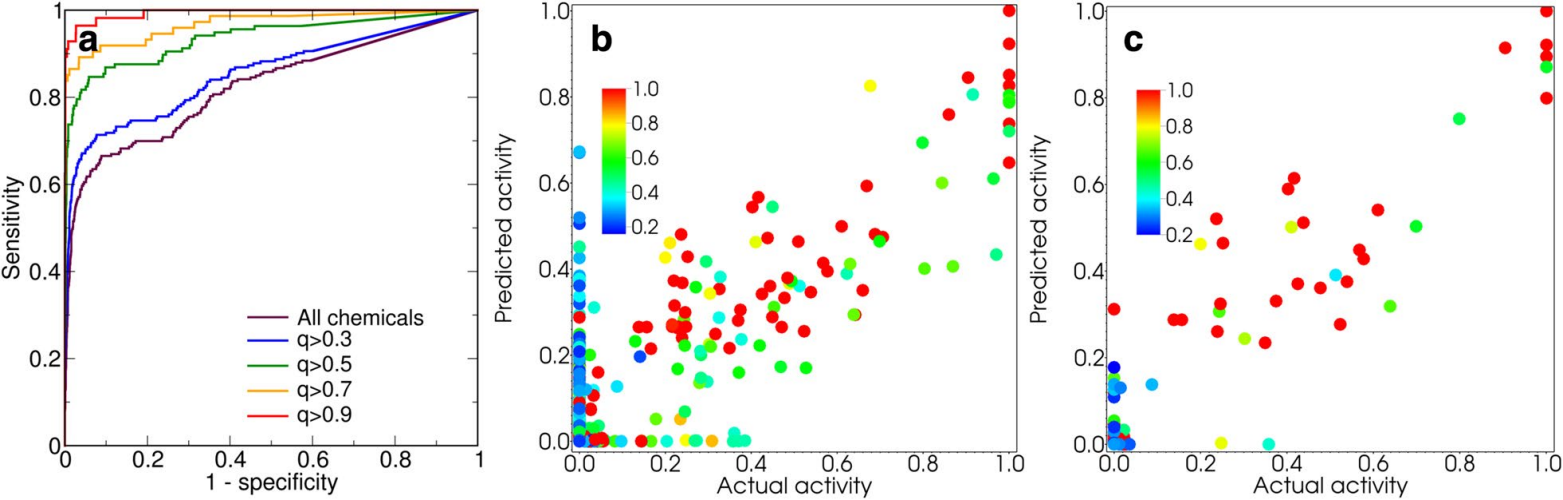
Performance of the GkNN model. Panel **a** ROC curves for the estimates of agonist activity of the evaluation set chemicals at different confidence values. Panels **b**, **c** agonist activities of the evaluation set chemicals estimated using the GkNN model versus those obtained from literature with more than 3 sources and more than 7 sources per chemical, respectively. Color indicates confidence level for each estimate

The dependence of the model performance on the confidence level of activity estimates $q_{i}$ is illustrated by Additional file 1: Table S2. For agonist activity, balanced accuracy and ROC AUC for chemicals with higher confidence levels are consistently higher than those calculated for chemicals with lower confidence levels. Panel A in Fig. 3 illustrates the dependence of ROC curves on confidence level, supporting the earlier suggestion that confidence levels can be used to define applicability domains for QSAR models.

For agonist activity estimates, the exponential kNN model and the GkNN model closely trails the CERAPP consensus model [16]. For antagonist activity, the exponential kNN model and the GkNN model consistently outperform the CERAPP consensus model for all estimates except those with $q \ge 0.9$. Since the training set included much fewer antagonist chemicals (9) than agonist chemicals (80), these observations reinforce the suggestion that employing non-linear distance metrics in the structure–activity space may be particularly efficient when training set data are limited. The influence of the uncertainty in the data from literature on the performance of the kNN models, the GkNN model, and the CERAPP consensus model is summarized in Additional file 1: Table S3 and illustrated in panels B and C in Fig. 3. As expected, for either model, increasing the number of literature sources for the evaluation chemicals (and thereby the quality of the activity data) results in increasing accuracy of the estimates and decreasing the number of false positive estimates, as illustrated in Additional file 1: Fig. S5.

## Conclusions

We introduced the GkNN QSAR model based on a custom non-linear distance metric in the chemical structure—biological activity space and explored how this non-linearity influences the model performance. Using the hER data from the ToxCast [9] and Tox21 [10] databases, we compared the accuracy of the GkNN model against that of other variants of the kNN model with non-linear weighting schemes and the CERAPP consensus model [16]. We found that the GkNN model, along with the exponential kNN model [19], appears most efficient when the training set data, most notably the number of active chemicals, are limited.

In this proof-of-concept study, we focused solely on the effects of the distance metric non-linearity and did not attempt to fully optimize the GkNN model. The latter can be achieved in multiple ways, for example, by optimizing the non-linear functions in the distance metric. Combining these steps with conventional approaches such as feature selection [8] may further improve the accuracy of QSAR models.

## Additional file

**Additional file 1.** Supporting information.

## Abbreviations

QSAR: quantitative structure–activity relationship
kNN: k-nearest neighbor (model)
GkNN: generalized k-nearest neighbor (model)
hER: human estrogen receptor
CoMFA: comparative molecular field analysis
CERAPP: collaborative estrogen receptor activity prediction project
PCA: principal component analysis
SALI: structure–activity landscape index
ROC AUC: receiver operating characteristics area under curve
