# Supplementary material for: Exploring non-linear distance metrics in the structure–activity space: QSAR models for human estrogen receptor
Source: J Cheminform. 2018 Sep 18;10:47. doi: 10.1186/s13321-018-0300-0 (PMC6755572; doi:10.1186/s13321-018-0300-0)
Supplement: Supplementary file 1 — Additional file 1. Supporting information. [file 13321_2018_300_MOESM1_ESM.zip › supporting_figures.docx]

**Supporting Information for “Exploring Non-Linear Distance Metrics in the Structure-Activity Space: QSAR Models for Human Estrogen Receptor”**


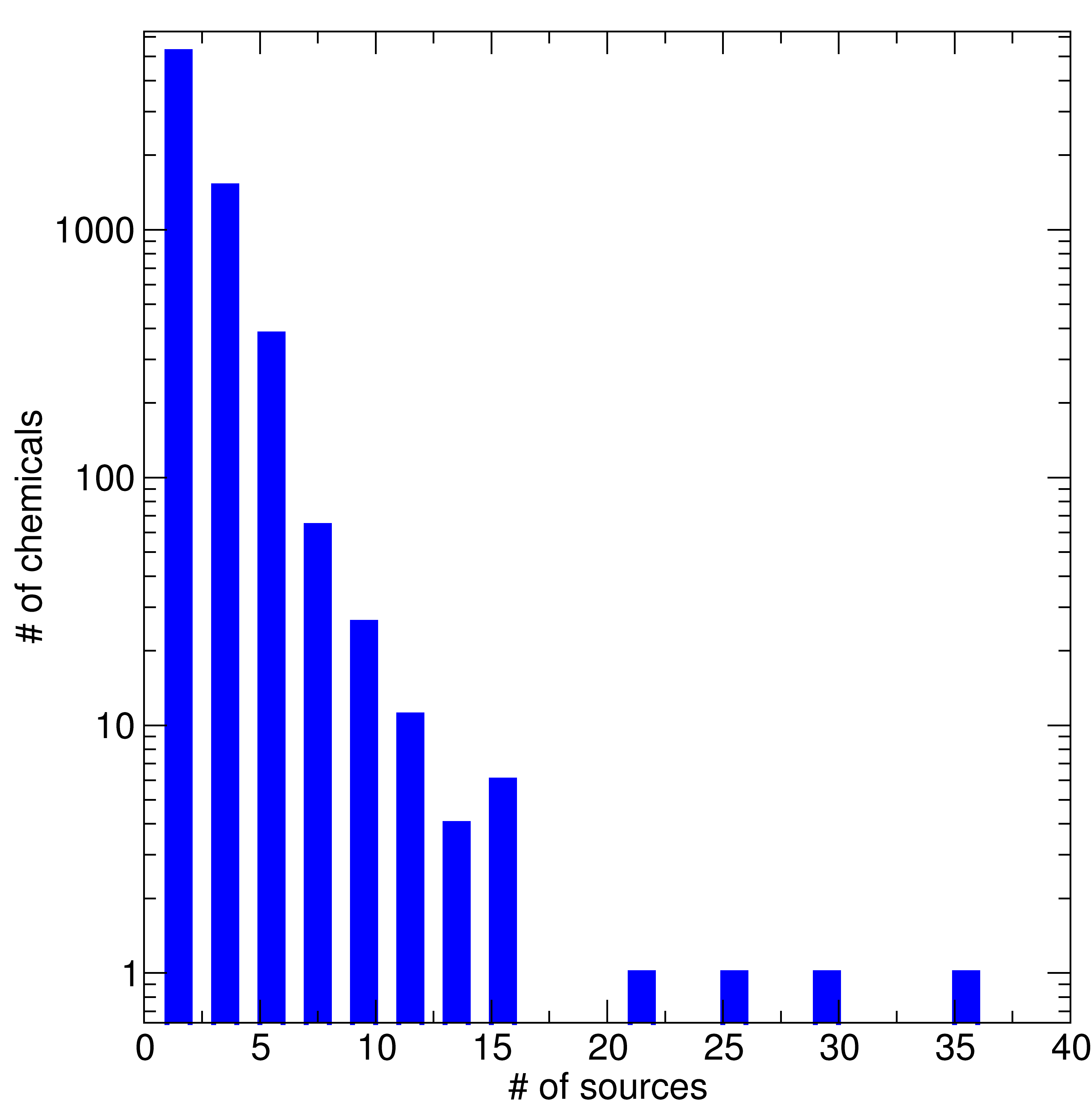


**Figure S1.** Number of chemicals in the evaluation set *vs.* number of their activity sources from literature.


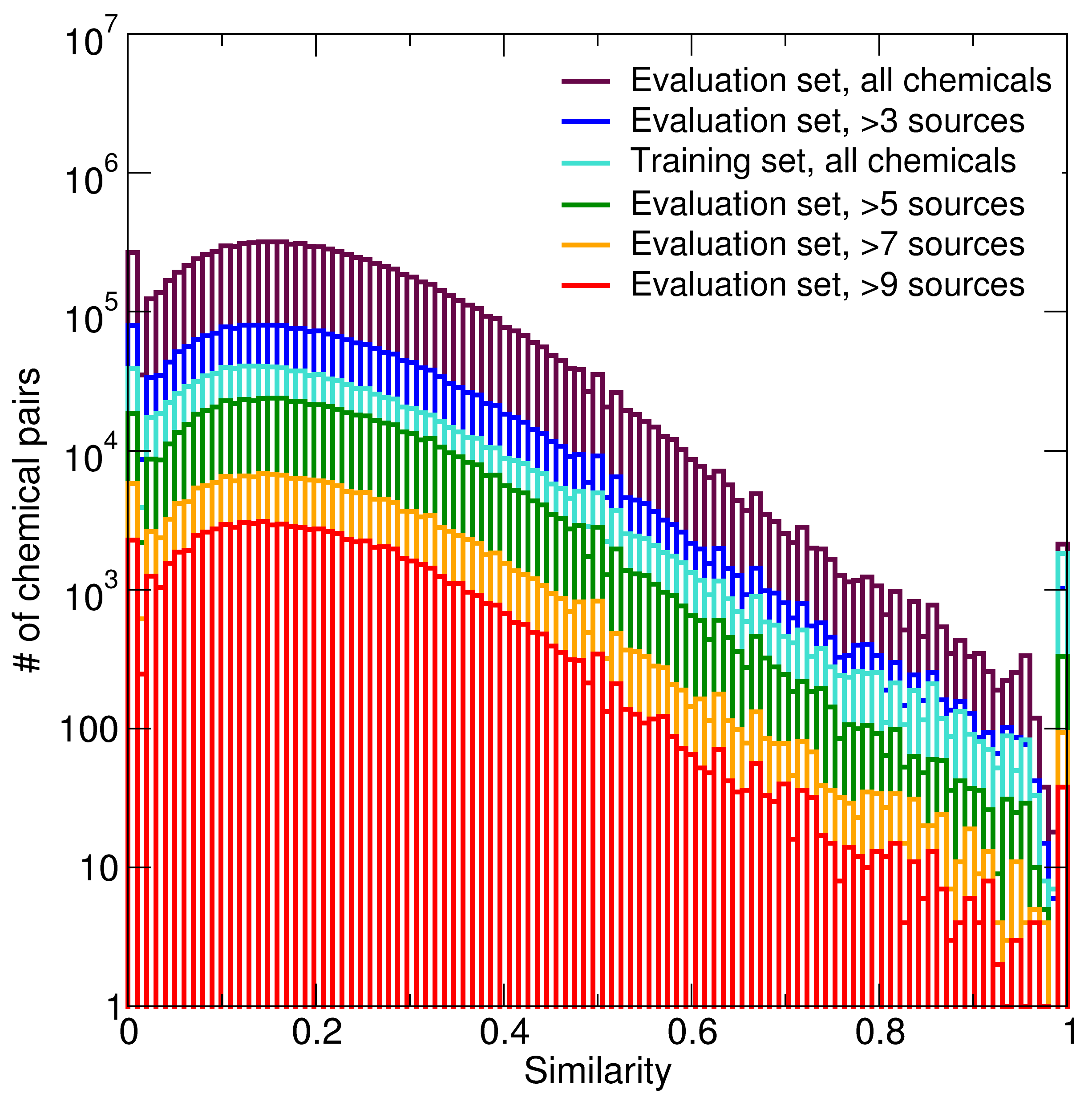


**Figure S2.** Distributions of pairwise molecular similarities calculated using MACCS keys (overlaid plots).


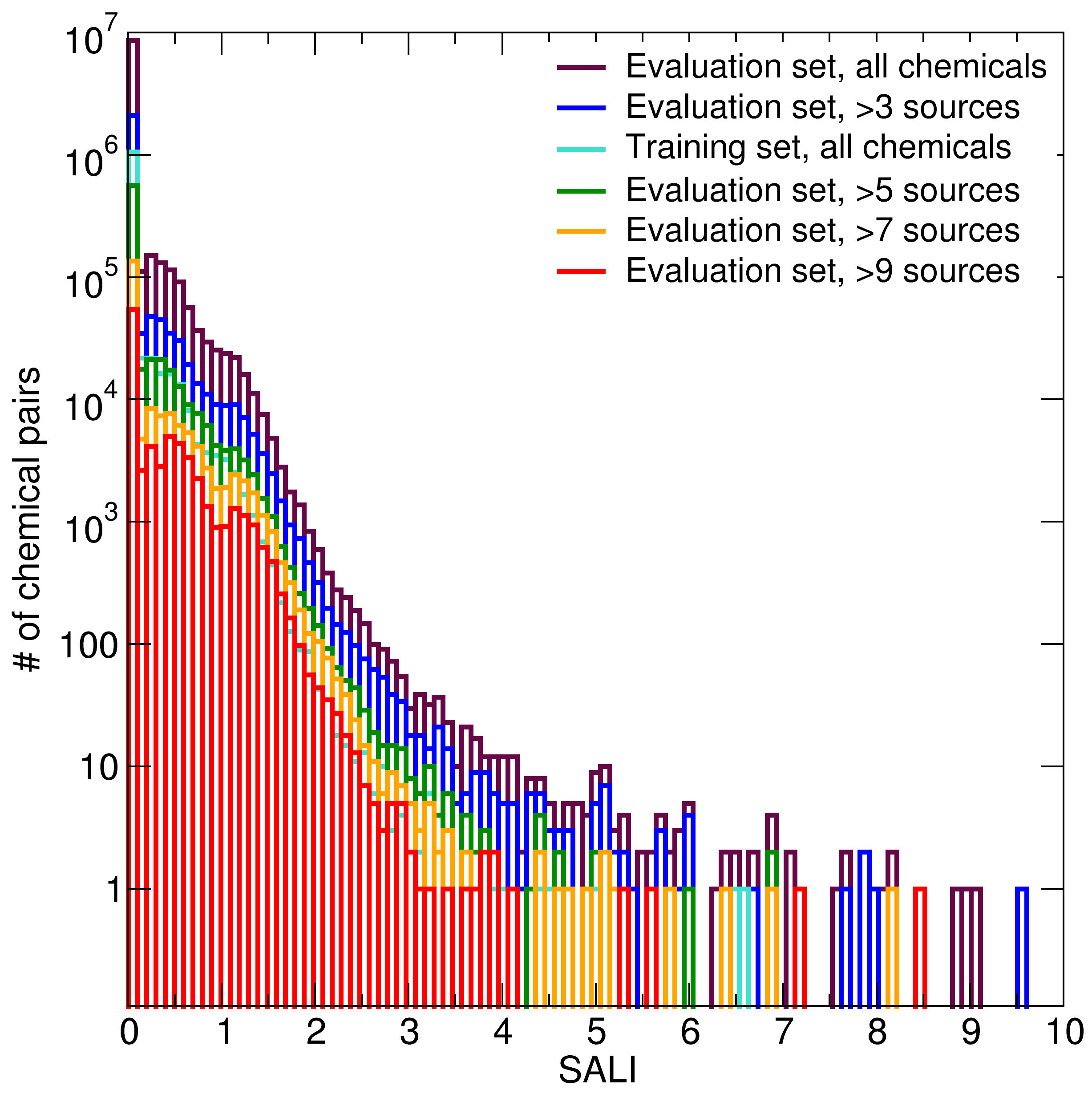


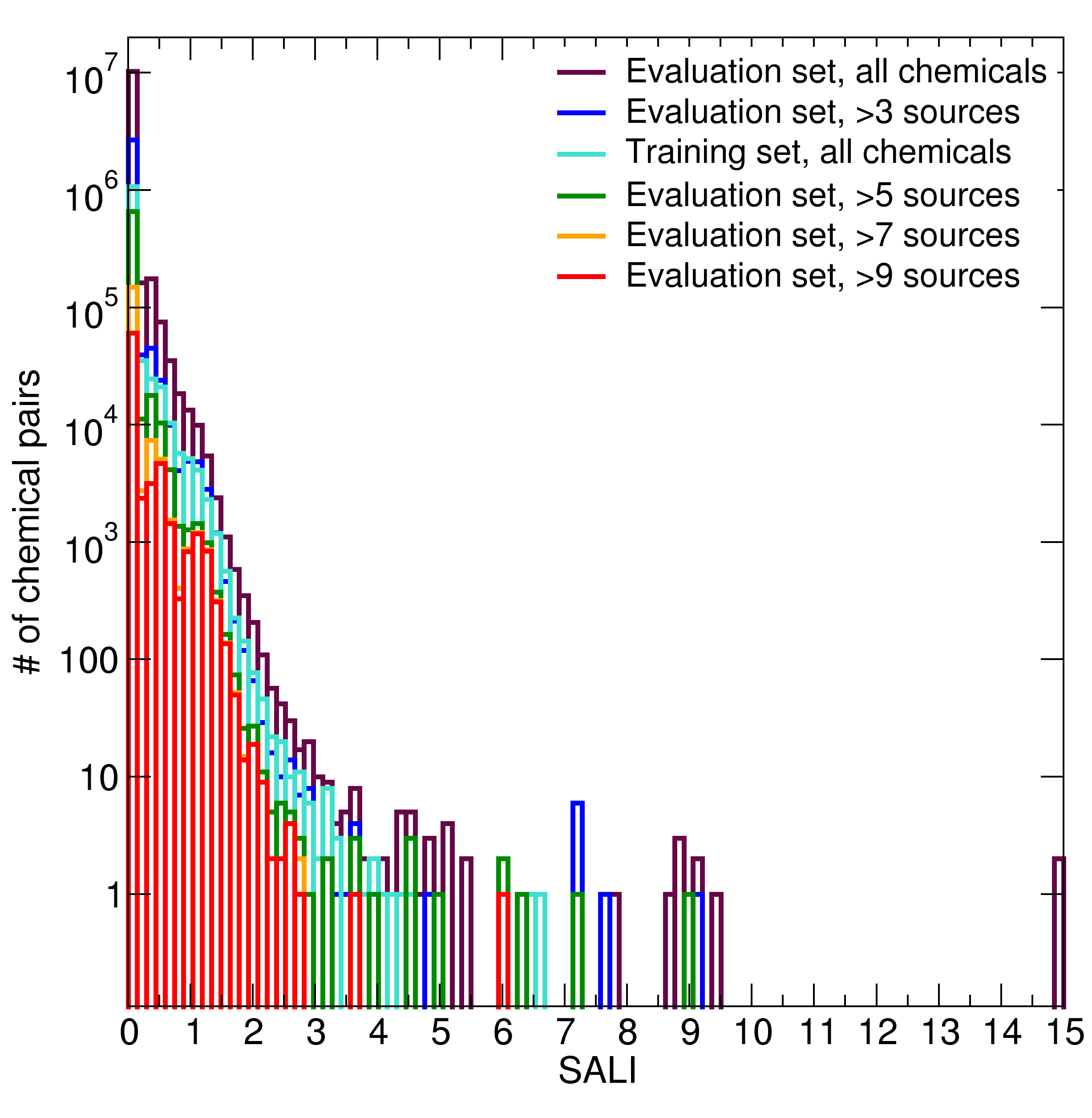


**Figure S3.** Distributions of SALI values for agonist activity (top panel) and antagonist activity (bottom panel) calculated using MACCS keys (overlaid plots).


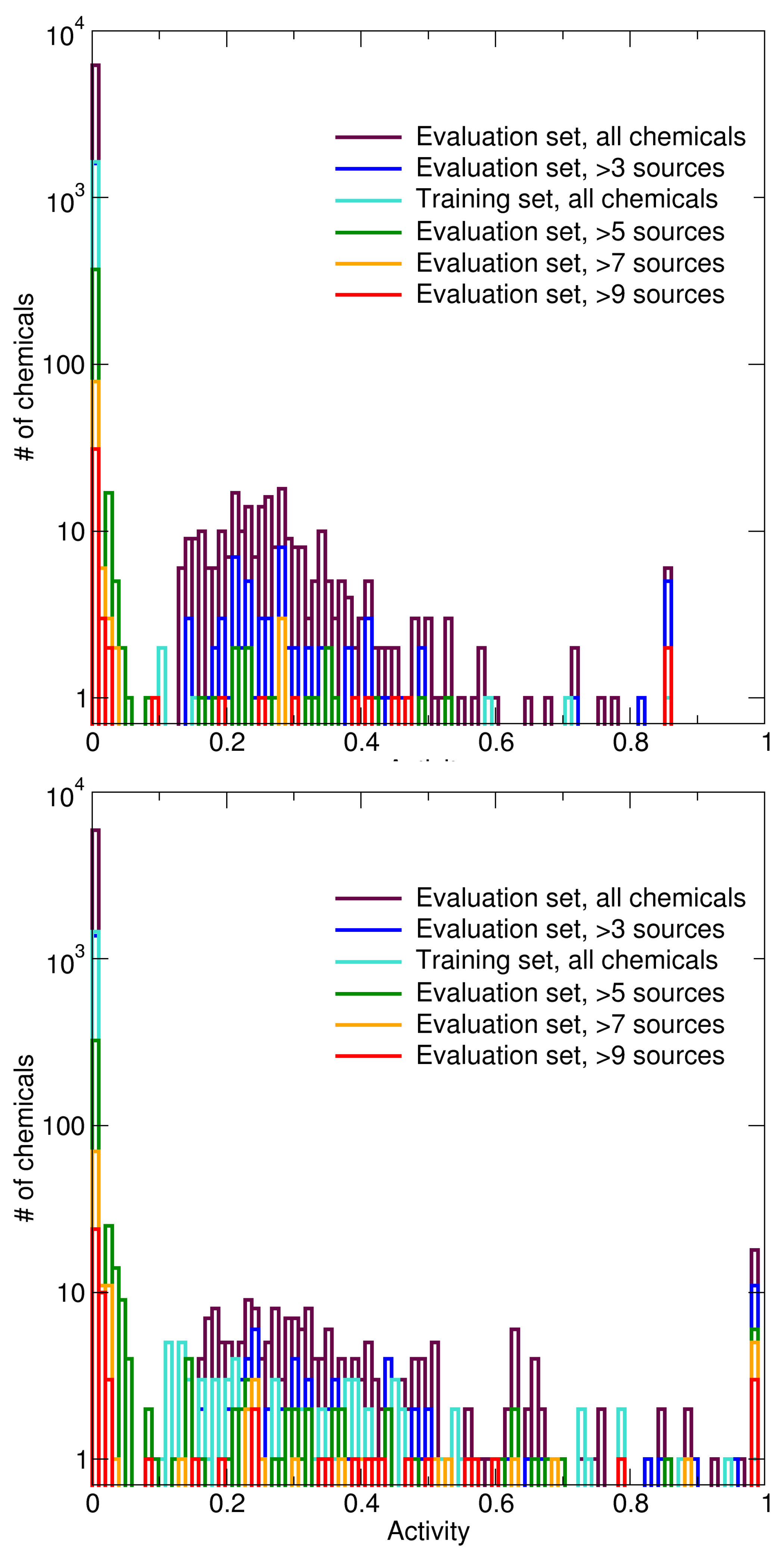


**Figure S4.** Distributions of agonist activity for chemicals in the training and evaluation chemical sets and subsets of the evaluation set (overlaid plots). Top panel: agonist activity; bottom panel: antagonist activi


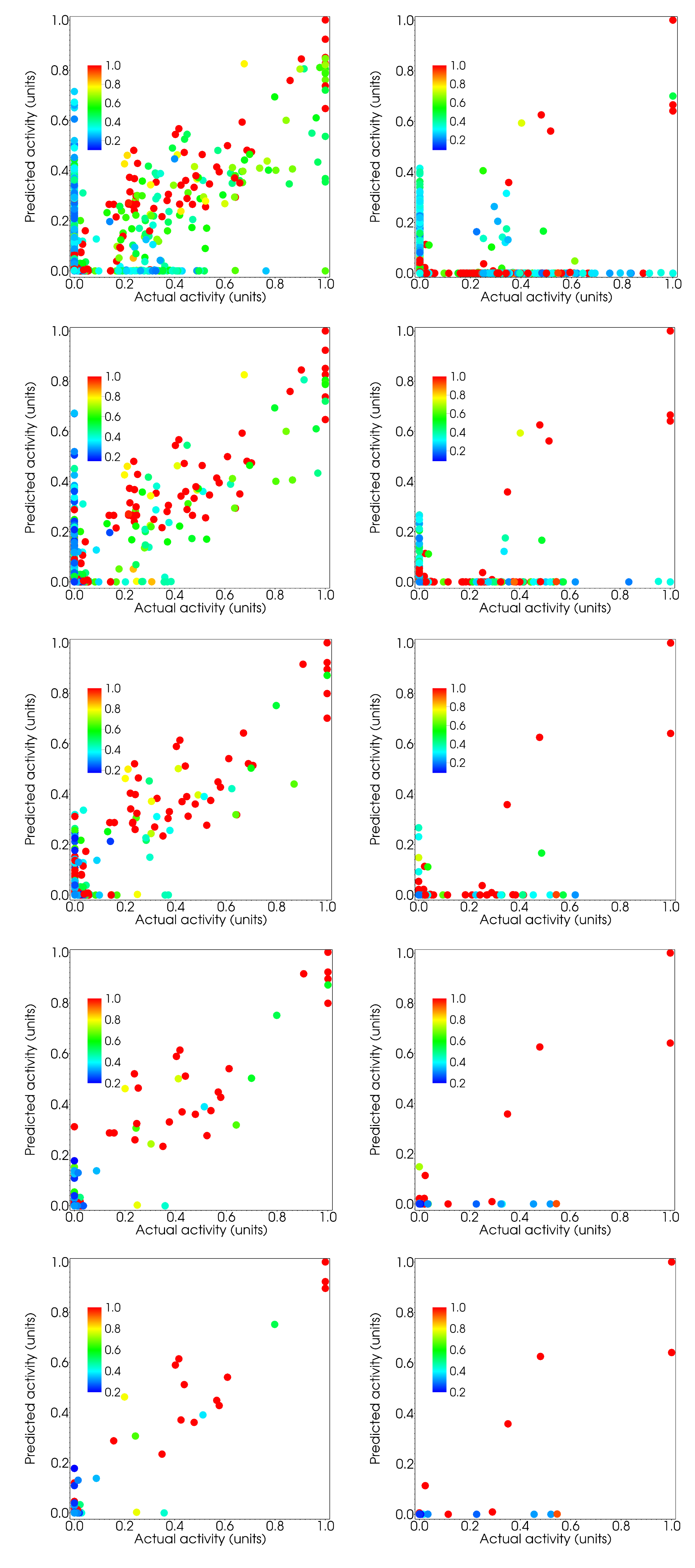


**Figure S5.** Estimated *vs.* obtained from literature agonist (left column) and antagonist (right column) activities of chemicals from the evaluation set. Top row shows all chemicals; the following rows include chemicals with more than 3, 5, 7, and 9 literature sources, respectively. Circle color indicates confidence in the estimate.
